# Optimising clinical trial methods for complex regional pain syndrome: a methodological framework (OptiMeth-CRPS)

**DOI:** 10.1097/PR9.0000000000001320

**Published:** 2025-10-03

**Authors:** Keith M. Smart, Victoria Abbott-Fleming, Frank Birklein, Stephen Bruehl, Erica Corcoran, Simon Day, Michael C. Ferraro, Sharon Grieve, Ralf-Dieter Hilgers, Carolyn Ingram, David J. Keene, Franz König, Candida McCabe, Stavros Nikolakopoulos, Neil E. O'Connell

**Affiliations:** a UCD School of Public Health, Physiotherapy and Sport Science, University College Dublin, Dublin, Ireland; b Physiotherapy Department, St. Vincent's University Hospital, Dublin, Ireland; c UCD Centre for Translational Pain Research, University College Dublin, Dublin, Ireland; d Burning Nights CRPS Support, Derbyshire, United Kingdom; e Department of Neurology, University Medical Centre, Mainz, Germany; f Department of Anesthesiology, Vanderbilt University Medical Center, Nashville, TN, USA; g Chronic Pain Ireland, Carmichael Centre, Dublin, Ireland; h Clinical Trials Consulting and Training Limited, North Marston, Buckinghamshire, United Kingdom; i Centre for Pain IMPACT, Neuroscience Research Australia, Sydney, Australia; j School of Health Sciences, Faculty of Medicine and Health, University of New South Wales Sydney, Sydney, Australia; k Royal United Hospitals Bath NHS Foundation Trust, Combe Park, Bath, United Kingdom; l School of Health and Social Wellbeing, University of the West of England, Blackberry Hill, Bristol, United Kingdom; m Institute of Medical Statistics, RWTH Aachen University, Aachen, Germany; n Faculty of Health and Life Sciences, Exeter Medical School, University of Exeter, Exeter, United Kingdom; o Center for Medical Data Science, Institute for Medical Statistics. Medical University of Vienna, Vienna, Austria; p Department of Psychology, University of Ioannina, Ioannina, Greece; q Biostatistics and Data Science, Julius Center, University Medical Center Utrecht, Utrecht, the Netherlands; r Department of Health Sciences, Centre for Wellbeing Across the Lifecourse, Brunel University London, United Kingdom

**Keywords:** Clinical trial, Complex regional pain syndrome, Methodological framework

## Abstract

**Introduction:**

Complex regional pain syndrome (CRPS) is a rare, disabling pain disorder. Systematic reviews have identified a critical lack of high-quality clinical trial evidence to inform the management of CRPS. There is an urgent need to find solutions to the methodological challenges of undertaking clinical trials in CRPS. The aim of this project was to develop a methodological framework for optimising future clinical trials in CRPS (OptiMeth-CRPS).

**Methods:**

An international network with expertise in the lived experience of CRPS, CRPS trials and evidence synthesis, and rare disease trial methods developed the framework using an “Experience and expertise” approach through an iterative process of (1) online and face-to-face meetings, (2) reviewing and approving meeting notes, and (3) revising draft manuscripts to develop the framework.

**Results:**

The OptiMeth-CRPS methodological framework presents 9 key optimisation strategies for improving the methodological quality of CRPS trials. These include strategies for optimising (1) the trial team, (2) research questions, (3) trial governance and management, (4) trial design, (5) the trial population, (6) intervention and comparator groups, (7) trial outcomes, (8) data analysis, and (9) openness, transparency, and reporting. We highlight those optimisation strategies that specifically address the challenges of undertaking clinical trials in people with CRPS.

**Conclusion:**

A methodological framework for optimising clinical trials in CRPS has been developed. The OptiMeth-CRPS methodological framework may support the CRPS research community to undertake high-quality clinical trials and improve the quality of the evidence on which clinical decisions and guidelines for the management of CRPS are based.

## Abstract 2

Supplemental Digital Content is Available in the Text.

The OptiMeth-CRPS methodological framework presents strategies for optimising the quality of clinical trials of interventions for Complex Regional Pain Syndrome as a rare pain condition.

## 1. Introduction

Complex regional pain syndrome (CRPS) is a painful and disabling condition that usually occurs in a limb after acute trauma, surgery, or spontaneously.^28^ Diagnosis is based on a cluster of characteristic symptoms and signs, known as the “Budapest criteria.”^48^ Population estimates suggest an incidence of somewhere between 5 and 26 cases per 100,000 person-years,^73^ as such CRPS is a rare condition.^28^

Current understanding of the pathophysiology of CRPS implicates multiple complex mechanisms linked to inflammation and autoimmunity, vasomotor dysfunction, central nervous system alterations, genetic susceptibility, and psychological distress.^28^ Living and coping with CRPS is challenging. It can have a far-ranging adverse impact on health-related quality of life, and the physical and social disability associated with living with CRPS persists in the long term for some sufferers.^57,58,70,82^ Emerging evidence suggests that a genetic predisposition in combination with an environmental trigger may contribute to the development of CRPS.^11,93^

Guidelines for the treatment of CRPS recommend an interdisciplinary multimodal approach, comprising rehabilitative, psychological, educational, pharmacological, and interventional pain management strategies.^39,47^ However, determining the optimal approach to therapy remains uncertain despite the availability of numerous clinical trials.^29^

Cochrane overviews^29^ and systematic reviews^79,81,97^ have identified a critical lack of high-quality evidence underlying most interventions for CRPS. This is due, in part, to the rarity of CRPS, variability in clinical presentations, and the associated challenges of recruiting and retaining sufficient numbers of participants but also to inadequacies in basic aspects of trial planning, design, conduct, and dissemination. Clinical trials involving people with CRPS are often characterised by sampling limitations (small sample sizes, single-centre recruitment), diverse outcome measures, and short-term follow-up periods. Furthermore, they often lack pre-registration, have no published protocol, and are incompletely reported.^47,97^ Improperly planned, designed, conducted, and reported clinical trials contributes to the waste of valuable research resources.^56^

In the absence of high-quality evidence supporting CRPS interventions, making treatment decisions and recommendations is extremely challenging for clinicians, clinical guideline developers, and people living with CRPS. Consequently, there is an urgent need to find solutions to the methodological and practical challenges of undertaking clinical trials in a rare chronic pain condition such as CRPS. Potential solutions could arise from optimising scientific quality and rigor throughout the clinical trial lifecycle, from ideation to dissemination,^72^ including optimising planning, designing, conducting, and reporting processes to enhance internal and external validity.^59^ Additional solutions could come from optimising methodological, statistical, and operational trial efficiency.^108^ An efficient trial is one that answers the research question robustly and accurately using the fewest resources. Achieving efficiencies in clinical trials in general and rare conditions such as CRPS specifically is highly desirable given the limited availability of human, economic, and material resources.

There are currently no CRPS-specific methodological frameworks aimed at improving the scientific quality of clinical trials of interventions for CRPS. A methodological framework that optimises trial methods may enable CRPS trialists to better fill the evidence void and in doing so, enhance the quality of the evidence upon which clinical guidelines and care are based.

### 1.1. Project aim

The primary aim of this project was to create a methodological framework that optimises the scientific quality of future clinical trials investigating the effects of interventions for people living with CRPS. For the purpose of this project, “scientific quality” refers to optimal practice in the planning, design, implementation, and dissemination of clinical trials.^59^

## 2. Methods of methodological framework development

### 2.1. Study registration

This project was registered on the Open Science Framework (OSF) (https://doi.org/10.17605/OSF.IO/894MQ). Ethical approval was not required for this project.

### 2.2. Project design

We used an “Experience and expertise” approach to develop a methodological framework.^75^ A methodological framework “provides structured practical guidance or a tool to guide the user through a process.”^75^ An experience and expertise approach uses the collective knowledge and experience of a group of experts to identify the issues and topics to inform and shape the framework and then iteratively develop the framework by synthesising and amalgamating the documented discussions of the group.^75^

### 2.3. Setting

The project was coordinated from University College Dublin, Ireland, by the project lead (KS). Three online (using a video conferencing platform) and two 2-day meetings (hosted in University College Dublin) were held between July 2023 and May 2024.

### 2.4. Participants

The methodological framework group comprised 14 purposefully sampled individuals based on their knowledge and expertise in (1) the lived experience of CRPS and/or patient advocacy (VAF, EC), (2) CRPS clinical trials (FB, SB, MCF, CM, NEO), (3) orthopaedic clinical trial methods and management (DJK), (4) CRPS clinical guidelines (FB, SB, SG, CM), (5) CRPS core outcome set development (FB, SB, SG, CM), (6) CRPS-related evidence synthesis (KS, MF, NEO) or (7) rare disease methodology and biostatistics (SD, R-DH, FK, SN). One project assistant (CI) compiled meeting notes.

Of the 15 members, 6 were based in the United Kingdom (CM, SG, VAF, SD, DK, NEO), 3 in Ireland (KS, CI, EC), 2 in Germany (FB, RD-H), and one each in Australia (MVF), Austria (FK), Greece (SN), and the United States of America (SB).

### 2.5. Procedure

Five meetings, chaired by the project lead, were scheduled to provide sufficient time and opportunity for the group to propose and discuss methodological issues and generate the framework. We used an iterative process of (1) online and face-to-face meetings, (2) reviewing and approving meeting notes detailing the group's discussions, and (3) draft manuscript revisions to develop the framework. Group discussions focused on optimising trial methods for CRPS as a rare multidimensional pain condition.

### 2.6. Deviations from protocol

The use of the Nominal Group Technique was not required to develop the final framework, which was achieved instead through group discussions, reviewing, and approving meeting notes and revising draft manuscripts.

## 3. Results

**Figure 1.**
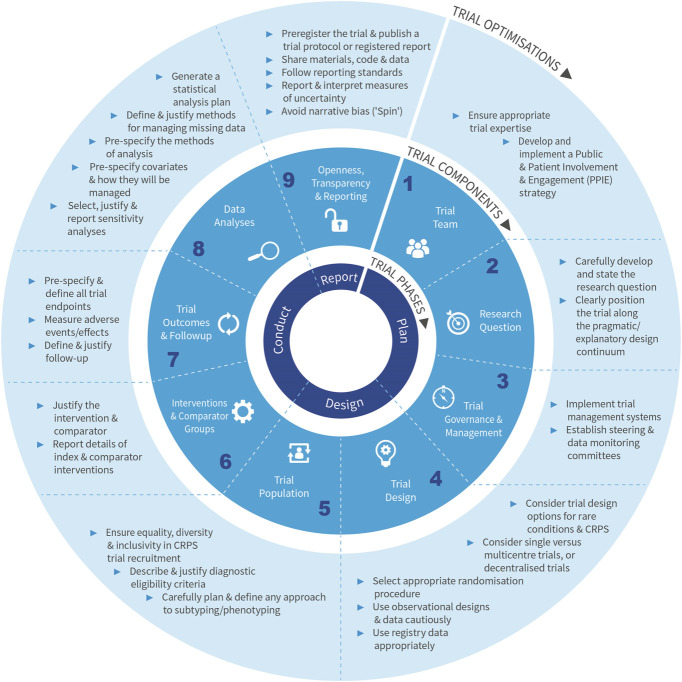
A summary of the OptiMeth-CRPS methodological framework.

The OptiMeth-CRPS methodological framework presents 9 key optimisation strategies for improving methodological rigor and efficiency spanning the planning, design, conduct, and reporting phases of CRPS trials. These include strategies for optimising (1) the trial team, (2) research questions, (3) trial governance and management, (4) trial design, (5) the trial population, (6) the intervention and comparator groups, (7) trial outcomes, (8) data analysis, and (9) openness, transparency, and reporting. We acknowledge the significant overlap and interrelatedness between trial components and phases. A summary of the overall framework is presented in Figure 1.

In this article, we highlight optimisation strategies within the methodological framework that specifically address the challenges of undertaking clinical trials in people with CRPS. However, as we considered the nature of the methodological limitations of many existing trials of interventions for CRPS,^24,79,81,97^ we identified opportunities for optimising a range of fundamental (eg, following reporting standards), contemporary (eg, enhancing Equality, Diversity, and Inclusiveness), and rare disease (eg, selecting a trial design) aspects of CRPS trials in addition to those specific to CRPS itself. We strongly recommend that readers consider the CRPS-specific optimisation highlighted in this article alongside the additional optimisation strategies reported in a freely available online white paper which details the complete OptiMeth-CRPS methodological framework.^98^ The meeting notes detailing the discussions at each meeting are also available online (https://doi.org/10.17605/OSF.IO/894MQ).

### 3.1. Optimising public and patient involvement and engagement

We recommend that CRPS trialists develop and implement a public and patient involvement and engagement (PPIE) strategy for including people with lived experience of CRPS and CRPS-advocacy groups within their trial teams to facilitate research meaningful to those living with CRPS. People living with CRPS and their representatives can valuably contribute their expertise and experiences to CRPS trial design (eg, formulating the research question, prioritising outcomes of interest), conduct (eg, advising on recruitment and retention), and dissemination (eg, cowriting plain language summaries).^4^ The Initiative on Methods, Measurement, and Pain Assessment (IMMPACT) group proposes 15 recommendations for enhancing PPIE in pain research relevant to CRPS trials.^51^

Complex regional pain syndrome trialists should consider the specific challenges of pain and mobility faced by PPIE contributors living with CRPS when deciding the nature, place, and timings of engagement. We encourage CRPS trialists to agree early with their PPIE partners, and remain flexible, on the scope of involvement, include them on trial steering/management committees, and ensure that their inclusion and participation is adequately resourced in funding applications and trial plans. Public and patient involvement and engagement has been successfully implemented in CRPS-related research to co-create an infographic to help support people living with CRPS,^7^ develop a core outcome set,^44^ and inform trial design and conduct.^41^ “Guidance for Reporting Involvement of Patients and the Public” (GRIPP2)^99^ in research is available.

### 3.2. Optimising the specificity of the research question in complex regional pain syndrome trials

The research question critically informs subsequent trial design and methodological decisions.^19^ Poorly focused or underdeveloped research questions may compromise the internal and external validity of a clinical trial.^27^ Therefore, CRPS trialists should carefully and clearly formulate their research question (and subsequent hypotheses, aims, and objectives) a priori, to focus the trial's purpose, make clear distinctions between exploratory (hypothesis generating) and confirmatory (hypothesis testing) trials, and express the hypothesised relationships between the variables under investigation.^27^ For CRPS trials, specifically this could include framing the research question according to the aim of the trial (eg, demonstrating superiority or noninferiority), specifying the clinical characteristics of the CRPS population of interest (eg, acute and/or chronic presentations, upper and/or lower limb),^40,44^ and stating the primary outcome(s) of interest (eg, pain intensity, function, or CRPS Severity Score [CSS]^49^).

### 3.3. Optimising trial design in complex regional pain syndrome trials

Systematic reviews of interventions for CRPS have demonstrated use of both parallel and crossover trial designs.^79,81,97^ Our group considers that most situations will call for conventional parallel trial designs. Alternative trial designs provide distinct opportunities to achieve efficiencies; for example, by optimising enrolment (eg, decentralised trials; N-of-1 designs) or requiring fewer participants for the same level of statistical power (eg, crossover designs); by allowing trialists to test 2 or more interventions in a single trial (eg, factorial designs) or shortening the duration of the trial (eg, adaptive designs). Decisions about trial design ultimately stem from the research question and invariably involve trade-offs between the advantages and disadvantages of a given trial design and between the desired efficiencies and the resources available.^108^

Algorithms to assist selecting between trial designs specifically involving people with rare conditions and smaller populations have been described.^18,45^ These algorithms involve the selection of different trial designs based on a range of disease-, recruitment-, outcome- and intervention-related characteristics. We make no specific recommendations concerning trial design because the decision will be likely based on a multitude of factors (eg, available expertise, financial resources, research setting, and regulatory environment.) and are best determined by individual trial teams. We considered the advantages and disadvantages of the different trial designs within these algorithms and their applicability to CRPS trials (summarised in Table 1).

**Table 1:** Advantages and disadvantages of different trial designs for complex regional pain syndrome as a rare condition (adapted from Ref. ^18,45^).

Trial design	Main features	Advantages	Disadvantages	Applicability to CRPS trials
Parallel	Participants are randomised to one of 2 (or more) treatment groups	Comparatively simple to design and conduct Well-understood and accepted	Larger sample sizes can be required compared with other designs Typically, last longer and more costly to run than many other designs	Highly applicable. Probably provide the simplest, most robust estimate of between-group differences in outcomes
Factorial^62^	Participants are randomised to one of 4 treatment groups (2 × 2 factorial trial), ie, (1) Treatment A alone; (2) treatment B alone; (3) both treatments A and B; or (4) neither A nor B	Enables the evaluation of more than one intervention in the same trial Can be very efficient regarding required resources and sample size (eg, 2 × 2 trial is equivalent to 2 parallel trials requiring around twice the sample size)	More complex design; can be challenging to implement Requires and assumes the effects of the different active treatments are independent (ie, no interaction between the treatments). Where an interaction is expected and is of interest, it can be estimated using this trial design but inflates sample size requirements resulting in some loss of efficiency	May be applicable if independence of treatment effects can be adequately justified or accounted for in the design
Crossover	Participants receive both index and control interventions according to a randomly assigned treatment sequence	Guaranteed exposure to the index intervention may improve enrolment Participants act as their own control, balancing covariates and reducing variability Require smaller sample sizes	More suitable for trials involving chronic, stable conditions and interventions with quick-onset and short-lasting effects Assumes participants' health status is comparable at the start of each treatment period. Adequate washout period required before crossover to remove potential carryover effects from the initial intervention Typically, last longer which may increase attrition rates	May be applicable only if symptomatic and clinical stability of the CRPS sample can be reasonably expected; hypothesised treatment effects are short-lived and/or adequacy of the washout period can be assumed
N-of-1^94^	A single participant receives periods of treatment according to a randomized sequence of multiple crossovers between treatment and comparison groups (eg, A-B-A-B; where one period ‘‘A’’ is the index treatment and the other period ‘‘B’’ is a comparison treatment) (eg, control or no intervention)	Optimising treatment for an individual patient Guaranteed exposure to the index intervention may improve enrolment Participants act as their own control, balancing covariates and reducing variance (Individual) N-of-1 trials for several patients using the same protocol offer the opportunity to pool study results	Same as for crossover design Less useful for providing generalisable estimates of treatment effectiveness but meta-analysis of individual N-of-1 trials might be useful for estimating population effects (homogenous outcome measures required)	Same as for crossover design Might be useful for rare conditions such as CRPS, participants otherwise excluded from trials, (eg, children, people with comorbidities or on concurrent treatments), investigating subgroups responses to treatment
Randomised withdrawal	All participants initially receive the index treatment; nonresponders are withdrawn; responders are then randomised to continue treatment or receive placebo/control	Useful for investigating optimal duration of treatment (in patients who respond to the treatment) May increase statistical power for a given sample size	Treatment effects may be overestimated as only responders proceed to randomisation Limited generalisability as the study population is treatment responders only	Might be useful for people with chronic, stable CRPS symptoms; investigating subtypes of CRPS People with CRPS may be unwilling to be randomised to a placebo/control after experiencing benefit
Adaptive^22^	A family of trial designs allowing preplanned changes to an ongoing trial's design or statistical procedures in response to accumulating trial data without compromising the validity of conclusions	Can achieve efficiency by reducing the required sample size (eg, by dropping interventions or stopping early through meeting prespecified utility or futility margins before reaching the full target sample size)	Highly complex to design, implement and analyse Can be more resource intensive in the design and conduct phases Some designs may risk rejecting potentially efficacious/effective treatments Planning and budgeting challenging as final sample size can often be uncertain	Could be applicable to drug trials. Applicability to multimodal and nondrug trials unknown

Crossover trials (where participants receive both index and control interventions according to a randomly assigned treatment sequence) and N-of-1 trials (singular or in series) as a variant of multiple crossover trials^94^ are a viable and efficient option for trials involving symptomatically stable conditions with relatively short-term end points.^21,53^ However, we advise caution in the use of crossover designs in people with CRPS because the variability of CRPS symptoms and signs^65,90^ may result in period effects (when the effect of the same treatment received at 2 different periods is different for each period) as well as carryover effects (when the effect of the first treatment alters the effect of a subsequent treatment).^67^ In particular, crossover trials may not be suitable for more acute, and potentially changeable, presentations of CRPS. In addition, washout periods to negate carryover effects prolong participation and follow-up which may increase participant dropout rates.^18^ Such losses are important because each participant in a crossover trial acts as their own comparator, resulting in twice the information loss compared to a participant in a parallel trial.^45^

Factorial trial designs (where participants are randomised to different combinations of two or more treatment groups in a single study) can increase efficiency by allowing evaluations of more than one intervention in a single trial without increasing the required sample size, although this efficiency depends on the assumption of no interaction (ie, synergistic or antagonistic effects) between compared treatments.^62^ The assumption of independence is not plausible in all contexts, and if violated, estimates may be biased. Potential interactions can be accounted for in the trial design, but this inflates sample size requirements resulting in some loss of efficiency. We know of one registered ongoing CRPS trial using a factorial design.^5^

Randomised withdrawal designs involve all participants receiving the index treatment initially after which “nonresponders” are withdrawn, and responders are randomised to continue treatment or receive a placebo/control intervention.^18^ The limitations of this design are similar to those for crossover trials. They may also overestimate treatment effects as only responders proceed to randomisation, limiting the generalisability of findings. We know of one trial that used a randomised withdrawal design and included people with CRPS as part of a mixed trial population^3^ but none in CRPS exclusively.^78^

Adaptive trial designs (eg, Sequential Multiple Assignment Randomized Trials, multiarm multistage) are a newer family of designs that allow preplanned changes to an ongoing trial in response to accumulating trial data without compromising the validity of conclusions.^22^ Adaptation options are potentially numerous but can include revising the sample size requirements in response to inaccurate assumptions of study design parameters; stopping a trial arm early in response to sufficient evidence of efficacy, futility, or safety concerns; or changing the treatment allocation ratio to favour treatments indicating beneficial effects.^22^ We are not aware of any previous or ongoing CRPS trials using adaptive designs. Adaptive designs can be combined with other trial designs and each other.^45^ We strongly recommend CRPS trialists consult with an experienced trial biostatistician if considering using a more logistically and methodologically complicated adaptive design to confirm suitability and viability.

### 3.4. Optimising sample size opportunities and accessibility in complex regional pain syndrome trials

Multicentre trials may be preferable for a rare condition such as CRPS to help achieve sample size requirements, reduce risk of bias, and enhance the generalisability of findings.^92^ The International Research Consortium for Complex Regional Pain Syndrome^55^ provides a forum to facilitate research collaborations and multicentre clinical trials involving people living with CRPS. However, multicentre trials are invariably more challenging to conduct, coordinate, and manage; more resource intensive; and require careful protocol adherence, quality assurance, and data management processes.^17,20^

In addition, multicentre trials usually involve centre-stratified randomization and stratified analyses. Heterogeneity of the treatment effects between centres may influence overall trial findings and need to be investigated.^76^ In some cases, where the number of patients per centre is small, stratification by centre cannot be implemented and study results must be interpreted relying on the assumption of no heterogeneity of treatment effects between centres.

The aforementioned factorial CRPS trial^5^ also uses decentralised trial methods. In decentralised trials, aspects of recruitment, enrolment, informed consent, delivery of study interventions and data collection may be conducted at locations other than clinical trial sites, through telemedicine, mobile/local health care providers, or digital technologies.^2,101^ By enabling broader equity of access and reducing participant burden, especially for people living with a painful and disabling condition such as CRPS for whom hospital visits can be extremely challenging and expensive, decentralised trials may improve participant enrolment, engagement, and retention and, by extension, the quality of trial data and the accuracy of findings.

However, decentralised trials are associated with various safety, privacy, and scientific validity challenges.^101^ For example, because there are currently no validated self-report CRPS diagnostic screening measures, fully decentralised trials using telemedicine may necessitate modifications to how diagnostic eligibility criteria are applied (eg, trial participants submitting photographs or videos of their limb or involving a partner to help with temperature and sensory tests to support a CRPS diagnosis). Decentralised trials may also influence which outcomes can be measured or interventions tested. For example, use of outcome measures (eg, the CSS^49^ or serology for biomarkers) or interventions (eg, pharmacological agents or devices) that require in-person medical administration or supervision may not be suitable.

Registry-based randomised controlled trials (rRCTs) are pragmatic trials that use existing patient data from registries to facilitate clinical trial procedures such as recruitment and collection of outcome data.^95^ As a sampling efficiency, it is also possible to use observational/natural history data from registries to supplement or replace a control arm in a clinical trial,^105^ although this requires careful consideration and planning and is often based on a range of conditions (eg, data quality) and assumptions (eg, that predicted treatment effects are large in comparison with the effect of potential biases).^36^

A planned international clinical research registry for CRPS may provide data useful to CRPS trialists in the future.^43^ Depending on the type and quality of data available, observational and/or trial data from a CRPS registry could be useful to trialists when planning and conducting a clinical trial. For example, registry data may be useful for estimating parameters to inform sample size estimates and identifying appropriate and meaningful end points.^32^ CRPS registry data could also be helpful in generating hypotheses about CRPS subgroups which can then be tested in a prospective RCT. We are not aware of the use of observational/registry data in CRPS trials, and we recommend CRPS trialists consult guidance and frameworks for evaluating the quality of observational and registry data if using such data in the future.^1,50,104^

### 3.5. Optimising the trial population through complex regional pain syndrome diagnostic eligibility criteria

The Budapest criteria for CRPS^48^ are the international standard for CRPS diagnosis and should be used to standardise trial eligibility and comparability, although our group noted that reliable application of inclusion/exclusion criteria can be challenging in multicentre and/or international trials.^20^ In practice, the broader Budapest “clinical criteria” can be used in preference to the stricter “research criteria” to increase participant eligibility and recruitment potential.^20^ We discourage the use of outdated diagnostic labels (eg, reflex sympathetic dystrophy, causalgia, poststroke shoulder-hand syndrome) and criteria (eg, “Veldman” criteria).^29,97^

However, given the rarity of the condition, trialists might consider using modified CRPS diagnostic criteria, ie, “CRPS in partial remission” for people who previously but no longer meet the Budapest criteria but who have some but not all ongoing symptoms and signs.^28,40^ Relaxing eligibility criteria is an efficiency that allows CRPS trialists to expand the potential eligible population from which participants might be recruited and increases the likelihood of reaching sample size requirements. However, caution is required as doing so may increase sample heterogeneity and reduce comparability with trials using standard Budapest criteria. Decisions regarding the selection of diagnostic eligibility criteria could depend on where the research question is located on the pragmatic—explanatory trial continuum,^68^ where explanatory/efficacy trials (could an intervention work in ideal circumstances) typically require the use of more stringent diagnostic criteria to enhance internal validity whereas pragmatic/effectiveness trials (does an intervention work in everyday clinical practice) may use less stringent clinical criteria based on “real-life” clinical populations to enhance external validity.^34^

Our group acknowledged the tension that exists in deciding between eligibility criteria for a rare condition such as CRPS that, if too narrow, may exclude too many patients, or if too broad, may introduce heterogeneity into the study sample. Ultimately, trialists should clearly describe and justify their eligibility criteria to optimise replicability, and to allow the applicability and generalisability of findings to be appraised. The need for trialists to thoroughly describe the clinical characteristics of their CRPS sample (eg, affected limb, limb dominance, participation in work/studying, inciting event, diagnostic symptoms and signs present, location and duration of symptoms) has been highlighted^40,44^ because they are sometimes incompletely reported.^97^

Our group also noted a potential ethnic bias in CRPS diagnostic criteria (skin colour changes/asymmetry) given that the Budapest criteria do not account for differences in skin colour. Validation of CRPS diagnostic criteria in people with different skin colours should improve their inclusivity, reliability, and applicability.

### 3.6. Optimising subtyping/phenotyping in complex regional pain syndrome trials

Distinct subtypes (or phenotypes) of CRPS have been explored and described (eg, acute/chronic; warm/cold; dystonic/nondystonic) based on hypothesised variations in the pathophysiological mechanisms underlying its presentation.^65^ Different mechanistic subtypes of CRPS may potentially benefit from treatments known or hypothesised to target those mechanisms in an attempt to optimise treatment outcomes.^71,83^ For example, a warm (ie, more inflammatory) mechanistic subtype may require and respond better to anti-inflammatory–based interventions compared with a cold (ie, less inflammatory) subtype.^12,23^ However, evidence for the validity of subtypes of CRPS is not yet sufficient to justify their use in confirmatory (hypothesis testing) clinical trials.^65^

The IMMPACT group has provided specific recommendations for patient subtyping/phenotyping in clinical trials for chronic pain conditions in general based on a number of possible domains, including psychosocial factors, symptom characteristics, sleep patterns, responses to noxious stimulation, endogenous pain-modulatory processes, and response to pharmacologic challenges.^25^ The extent to which CRPS might reflect subtypes according to these domains is not currently known. We therefore encourage CRPS trialists with an interest in phenotyping and subgrouping to further investigate the validity of these subtyping domains using appropriately designed studies.^64^ For example, CRPS trialists might define subtypes and then analyse them as potential effect modifiers.^30^

We recommend that CRPS trialists follow appropriate methodological guidance if planning to conduct “subgroup” (synonymous with the subtype but without an implied shared mechanism) analyses.^9,13,26,30,46,64^

### 3.7. Optimising the intervention and comparator groups in complex regional pain syndrome trials

A recent overview of systematic reviews of interventions for treating pain and disability in adults with CRPS found that many included trials tested interventions against active comparators without prior evidence of efficacy using placebo control,^29^ suggesting that trialists may be moving to comparative effectiveness trials prematurely.

When planning future trials, we encourage CRPS trialists to systematically evaluate existing data on efficacy and effectiveness to justify the selection of their intervention(s), frame their research question, and inform intervention parameters (ie, components, dosage, mode of delivery). If such data are absent, CRPS trialists should undertake exploratory proof of concept/hypothesis generating studies in accordance with the intervention development and evaluation lifecycle.^42,96^ Such preliminary, intervention development studies are required to support the biological plausibility, feasibility, tolerability, acceptability, adherence, fidelity, safety, and potential scalability of prospective interventions before undertaking more complex and costly clinical trials.^6,84,85,91,100,109^

Frameworks are available to assist CRPS trialists when planning, developing, and evaluating early-, mid-, and late-stage CRPS interventions.^8,38,42,96^

Our patient insight partners highlighted the need for CRPS trialists to provide quality plain language information within participant information resources that more clearly distinguishes between trials investigating established (eg, pragmatic trials) in contrast to more novel or experimental (eg, mechanistic, exploratory trials) interventions. They also highlighted the need for CRPS trialists to consider, in partnership with patient representatives, the duration of comparator interventions as trial participants are unlikely to want to receive placebo interventions for protracted periods of time. This consideration may inform the choice of trial design because the duration of placebo periods varies between them.

Furthermore, systematic reviews of interventions for CRPS show that trialists do not always fully describe their index and comparator interventions.^81,97^ In response, CRPS trialists should fully report the details of their interventions in accordance with established guidelines.^54,^ Reporting the nature, known or hypothesised mechanisms of action, and parameters of trial interventions thoroughly is essential for enabling trial interpretability and replicability.

### 3.8. Optimising trial end points, reporting adverse events and follow-up in complex regional pain syndrome trials

We recommend that CRPS trialists select clinical end points informed by the Core Outcome Measurement Set For Complex Regional Pain Syndrome Clinical Studies (COMPACT).^44^ CRPS trialists should also consult with their own patient partner groups to ensure that the COMPACT are applicable to them and to consider other potential outcomes of interest. We appreciate that outcomes of interest will vary according to the trial's aims (eg, explanatory, pragmatic, mechanistic, feasibility). We also acknowledge the challenge of selecting one primary outcome for a complex and multidimensional condition such as CRPS (eg, changes in pain intensity vs function vs quality of life). Trial teams should therefore consider which dimension of the CRPS experience the intervention is targeting when choosing their primary end point. Our patient insight partners highlighted the importance of and need to measure quality of life (QoL) because QoL may improve when pain intensity does not.

For confirmatory trials of interventions for rare conditions such as CRPS, it may be advisable to avoid co-primary end points (when it is necessary to demonstrate “significant” effects on all prespecified end points to conclude that an intervention is effective), as the power (and efficiency) of a trial is normally reduced by the requirement to demonstrate significant effectiveness of more than one end point, unless those end points are highly correlated.^74^

Complex regional pain syndrome trialists using multiple primary end points (when it is necessary to demonstrate a “significant” effect on any one of a number of prespecified end points to conclude that an intervention is effective), should consider and report their methods for adjusting for multiple comparisons in the analysis.^102^ Options for handling multiple end points in general and rare disease clinical trials have been described and should be carefully considered.^33,88^

The definition and reporting of adverse events/effects (AEs) in CRPS trials are known to be inadequate, prohibiting robust evaluations of intervention safety.^29^ We recommend that future CRPS trialists plan (a priori) and report their methods for measuring AEs in accordance with relevant guidelines.^16,60^ For trials in people specifically with CRPS, this should include evaluations of potential withdrawal symptoms associated with pharmacological interventions and longer-term evaluations of implanted devices.

Follow-up time points for outcomes of interest, including safety, are likely to vary according to the clinical characteristics of the trial population (eg, acute or chronic) as well as the purpose of the study and the research question. We propose that the duration of follow-up should be informed by the nature of the intervention and its goals, and in collaboration with patient and clinical stakeholders. When trialling interventions that are predicted to have longer-term effects, our group recommends a minimum of 6 months follow-up.

### 3.9. Optimising methods of analysis and covariate selection in complex regional pain syndrome trials

A recently updated Cochrane systematic review of physiotherapy interventions for CRPS showed that the majority of trials either did not report their analysis method (53%) or violated the “Intention-to-treat” (ITT) principle (26%).^97^ Intention-to-treat, whereby participants are analysed according to the treatment group to which they were originally assigned, is the preferred approach to analysis because it maintains randomization (ie, comparability of groups at baseline with respect to measured or unmeasured prognostic factors).^52^ This suggests that some CRPS trialists could improve their application and reporting of ITT. The estimands framework may usefully help trialists specify their analysis strategy.^61^

Adjusting for baseline prognostic covariates (ie, measurable characteristics of a trial population that have a statistical relationship with the outcome variable) in the analysis of trials enhances statistical efficiency. Accounting for the variance in (continuous) outcomes explained by covariates reduces standard errors for the treatment effect and minimises the sample size required,^63^ an efficiency likely to be attractive to CRPS trialists. Selecting which covariates to include in the analysis of CRPS trials should be based on data from previous trials on similar patient populations or clinical observations of factors known or expected to have strong or moderate associations with the primary outcome.^66,86^ For pain trials in general, baseline prognostic covariates could include demographic (eg, age, sex, ethnicity, workplace compensation claims), pain (eg, pain intensity or duration), psychological (eg, depressive symptoms), or cognitive (outcome expectation) factors.^66^ The nature, extent of use, and associations of covariates in relation to common primary end points in CRPS trials are not known but should be systematically investigated. However, potential biological and psychological prognostic factors in recently diagnosed CRPS, based on moderate quality evidence, include baseline pain intensity, self-rated disability, anxiety, depression, catastrophising and pain-related fear, female sex, and a history of a high-energy triggering event.^10,69^ These could be considered as candidate baseline prognostic covariates by future CRPS trialists.

It is critical that covariates are prespecified for the primary analysis, appropriately justified, and not selected and adjusted for post hoc, which could compound the risk of false-positive conclusions.^66,86^ The number of covariates used should be limited relative to the usually small/modest sample sizes in CRPS trials. Including non-prognostic covariates may reduce trial power and has been discouraged.^63^

### 3.10. Optimising openness, transparency, and reporting

A recently updated Cochrane systematic review of physiotherapy interventions for CRPS found that 63% of trials conducted between 2015 and 2021 were either not pre-registered or associated with a published trial protocol.^97^ Pre-registration and protocol publication enhance transparency and credibility and likely reduce potential bias, arising from practices such as outcome switching (changing which outcomes to report or emphasise), p-hacking (analysing data to find statistically significant results), and HARKing (hypothesising after the results are known).^14^ Given the potential bias associated with unregistered trials and trials without published protocols, we strongly recommend that all future CRPS trialists register their trials and publish a trial protocol in accordance with recommended guidelines.^16^

Existing overviews and reviews of trials for CRPS^29,81,97^ demonstrate that methodological reporting guidelines^16,54,89^ are not consistently used. We recommend that CRPS trialists specifically plan their trials and report their findings in accordance with CONSORT guidelines relevant to their trial design (eg, factorial trials), methods (eg, use of patient-reported outcomes), types of data (adverse events), and intervention (eg, nonpharmacologic).^37,77^ A reporting and reviewing checklist specific to pain-focused clinical trials is also available.^35^ Following reporting guidelines provides the transparency necessary for others to (1) critically appraise and interpret findings, (2) replicate the trial, and (3) consider implementing its findings.^15^

Researchers have been encouraged to accept, measure, and communicate uncertainty.^104^ However, evidence syntheses show that CRPS trialists inconsistently report results, including effect sizes and statistical measures of uncertainty and precision (eg, standard deviation, confidence intervals, sensitivity analyses).^81,97^ Our group highlighted the need for CRPS trialists to fully report these data and interpret and communicate their findings in light of these uncertainties.^31^

## 4. Discussion

The OptiMeth-CRPS methodological framework presents a range of strategies for optimising the rigor and efficiency of clinical trials of interventions for CRPS across the planning, design, conduct, and reporting phases of the trial lifecycle. It addresses and offers solutions to many of the methodological challenges of undertaking clinical trials in people living with CRPS.

It reflects and builds upon evolving general,^106^ pain, and rare condition-based methodological knowledge and recommendations by providing clear flexible guidance that specifically addresses the challenges of undertaking clinical trials for CRPS as a rare pain condition. It is offered as a tool to support the CRPS research community to undertake high-quality clinical trial research to better guide clinical practice.

Uncertainties underlying the findings from many previous trials of interventions for CRPS arising from insufficiently planned, designed, conducted, and reported trials^29,81,97^ and from small sample sizes owing to the rarity of the condition indicate that the scientific quality and efficiency of trial methods could be improved. Methodologically flawed pain trials that do not meaningfully contribute to the evidence base waste valuable research resources, delay discovery and implementation of treatments, and may ultimately harm trial participants.^80^ It is not our intention to complicate or obstruct clinical trials for CRPS but to propose solutions to the numerous complexities and challenges of undertaking such trials to improve their rigor and value and reduce research waste. Our framework is aligned with the 2024 revision of the World Medical Association's (WMA) Declaration of Helsinki which states, “Medical research involving human participants must have a scientifically sound and rigorous design and execution that are likely to produce reliable, valid, and valuable knowledge and avoid research waste.”^107^

Although this methodological framework was developed primarily as an aid for CRPS trialists, it may also benefit peer reviewers and journal editors, funders of CRPS trials, CRPS clinical guideline developers, clinicians, and those with lived experience of CRPS when considering publishing, funding, supporting, or using the findings from future trials. Furthermore, because many of the methodological issues and challenges associated with undertaking clinical trials in CRPS as a rare pain condition are also applicable to pain trials, in general, this framework may be useful to the pain trial community more broadly.^87^

It remains to be seen whether and how this methodological framework is implemented by CRPS trialists and others. Methodological frameworks can be refined and validated by undertaking evaluations of their real-world utility.^75^ Evolving knowledge and understanding of general, pain, and rare condition trial methods together with any subsequent feedback from the pain, CRPS, and rare disease communities will likely necessitate the revision of this methodological framework in the future.

We have endeavoured to provide guidance based on the collective knowledge and expertise of an interdisciplinary international group of CRPS, rare condition methodology and biostatistics, evidence synthesis and patient experience experts; informed by and with reference to best practices. However, our article should be interpreted in light of a number of potential limitations. We acknowledge that there is no single best or standardised approach for developing methodological frameworks and that this article represents the collective opinions of one purposefully sampled group. A different, more geographically diverse group of individuals, using similar or different methods may have generated alternative perspectives, opinions, and recommendations. For example, our group did not consider the specific issues of (1) susceptibility to treatment side effects, (2) common comorbidities, and (3) issues with concomitant treatment use in CRPS trials.

It is our belief that optimising trial methods in CRPS will improve the quality of the evidence upon which clinical decisions and guidelines for the management of CRPS are based, and in doing so, optimise outcomes for people living with CRPS.

## Disclosures

KMS has received financial support from the European Pain Federation (EFIC) to attend Congresses of the European Pain Federation (EFIC, 2023). SB has received research grant funding through the US National Institutes of Health and is a consultant for Akigai. SD has received research grants from the European Commission and travel bursaries from the European Commission and National Institutes of Health. In addition, SD works as a consultant to the pharmaceutical industry. DJK's institution receives research grants from the National Institute for Health and Care Research (NIHR). NEO is a member of the Cochrane Central Editorial Board. Between 2020 and 2023, NEO was Co-ordinating Editor of the Cochrane Pain, Palliative, and Supportive Care group, whose activities were funded by an infrastructure grant from the UK National Institute of Health and Care Research (NIHR). He currently holds a networking grant from the ERA-NET Neuron Cofund. The remaining authors have no conflicts of interest to declare.

## Supplemental digital content

Supplemental digital content associated with this article can be found online at https://doi.org/10.17605/OSF.IO/894MQ.
